# Design, Synthesis and Evaluation of N13-Substituted Evodiamine Derivatives against Human Cancer Cell Lines

**DOI:** 10.3390/molecules181215750

**Published:** 2013-12-17

**Authors:** Senchuan Song, Zhiyong Chen, Shaoxue Li, Yanmin Huang, Yiqian Wan, Huacan Song

**Affiliations:** 1 School of Chemistry and Chemical Engineering, Sun Yat-sen University, Guangzhou 510275, China; 2 College of Chemistry and Life Science, Guangxi Teachers Education University, Nanning 530001, China

**Keywords:** N13-substituted evodiamine derivatives, antitumor activity, apoptosis, solubility

## Abstract

Attempting to improve the anticancer activity and solubility of evodiamine in simulated gastric fluid (SGF) and simulated intestinal fluid (SIF) solutions, thirty-eight N13-substituted evodiamine derivatives were designed, synthesized and tested for antitumor activities against six kinds of human cancer cell lines, namely prostate cancer (DU-145 and PC-3), lung cancer (H460), breast cancer (MCF-7), colon cancer (HCT-5) and glioblastoma (SF-268). The solubility of these compounds in SGF and SIF solutions was evaluated, and apoptosis induced by **2-2**, **2-3**, **2-16** and **3-2** was determined. The results showed: (1) among all compounds examined, **2-16** showed the highest antitumor activity and a broader spectrum of activity, with IC_50_ values ranging from 1–2 µM; (2) their solubility was obviously improved; (3) **2-3**, **2-16** and **3-2** had a significant impact inducing apoptosis in some cancer cell lines. The preliminary structure-activity relationships of these derivatives were discussed.

## 1. Introduction

**Figure 1:**
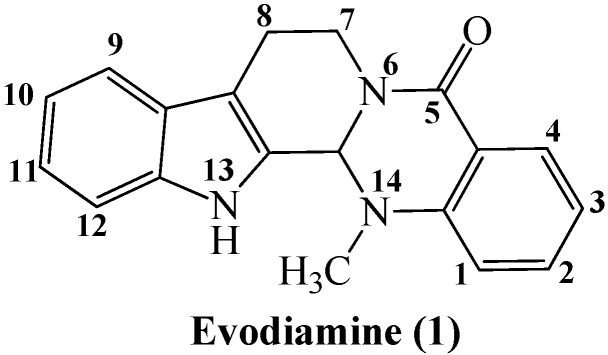
Chemical structures of evodiamine (**1**).

Evodiamine (**1**, Figure 1) is a quinolone alkaloid isolated from the fruit of *Evodia rutaecarpa* (Chinese name: Wu-Chu-Yu), which is one of the most popular and multi-purpose Traditional Chinese Medicines widely used for treating diverse human disorders [1] and is also very attractive as a component of healthy foods [2,3]. It was demonstrated by Hu [4] and Linag [1] that evodiamine possesses a wide range of biological activities related to anti-inflammatory, anti-obesity and antitumor properties. In particular, both the strong cytotoxicity of evodiamine against human cancer cells and the underlining mechanisms of growth inhibition, apoptosis induction and suppression of invasion and metastasis have attracted much attention [4,5,6,7,8,9,10,11,12,13,14,15,16,17,18,19,20,21,22]. Moreover, it was demonstrated that evodiamine sensitized chemoresistant breast cancer cells to adriamycin without obvious cytotoxicity against normal human peripheral blood cells [4,23], which indicated the potential of evodiamine for clinical application. In 2010, Sheng and his co-workers identified N13-substituted evodiamine derivatives to be potent topoisomerase I inhibitors by structure-based virtual screening and lead optimization [13]. Recently, the same group has constructed a library of new evodiamine derivatives bearing various substitutions or modified scaffold with substantially increased antitumor activity, which is attributable to the inhibition effect on topoisomerase I and II [16]. Despite its promosing anticancer potential, evodiamine is still unsuitable for clinical application because of its poor physicochemical properties.

In this study, we were interested in using evodiamine as a lead compound to develop anticancer drug candidates, because of its: (1) known broad-spectrum antitumor activities; (2) significant difference of toxicity between cancer cells and normal peripheral blood mononuclear cells; (3) good drug-like molecule features as judged by both the criteria of Lipinski’s “rule of five” and the “verb rule”; (4) good synthetic accessibility for its diversity of derivatives. In our continuous search for novel improved antitumor agents [24,25,26,27,28,29], we report herein the design, synthesis and testing of novel N13-substituted evodiamine derivatives as antitumor agents.

## 2. Results and Discussion

### 2.1. Synthesis of N13-Substituted Evodiamine Derivatives

**Scheme 1:**
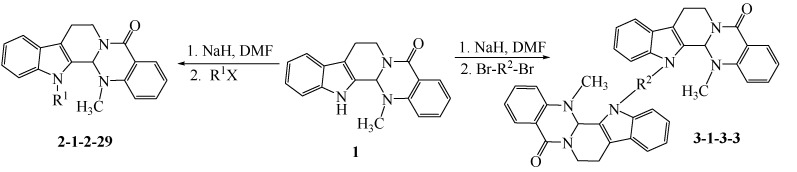
Synthesis of N13-substitued evodiamine derivatives **2-1**–**2-29** and **3-1**–**3-3**.

**Scheme 2:**
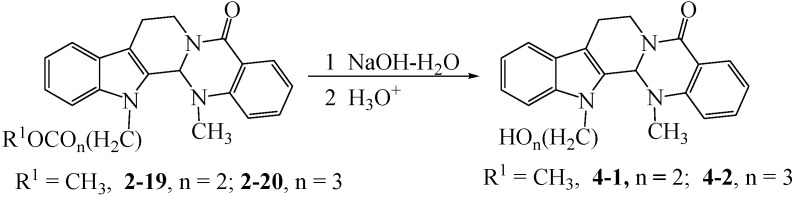
Synthesis of N13-(hydroxyalkyl)evodiamine derivatives **4-1** and **4-2**.

**Scheme 3:**
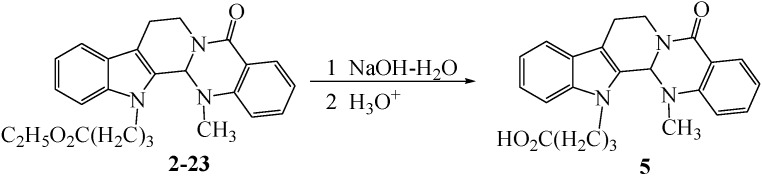
Synthesis of 4-(evodiamin-N13-yl)butyric acid **5**.

**Scheme 4:**
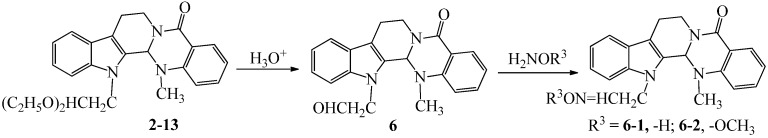
Synthesis of **6**, **6-1**–**6-2**.

The synthetic pathways to the target compounds are outlined in Scheme 1, Scheme 2, Scheme 3 and Scheme 4. The substitution reactions of evodiamine with alkyl halides or *p*-toluenesulfonic acid ester at the N13-position of evodiamine were completed smoothly in dried *N,N*-dimethylformaide (DMF) as reported in reference [13] with slight modifications.

R^1^ = **2-1**, -CH_2_CH=CH_2_; **2-2**, -(CH_2_)_3_CH_3_; **2-3**, -(CH_2_)_4_CH_3_; **2-4**, -CH_2_CH=C(CH_3_)_2_; **2-5**, -(CH_2_)_7_CH_3_; **2-6**, -(CH_2_)_3_C_6_H_5_; **2-7**, -CH_2_CH(CH_3_)_2_; **2-8**, -(CH_2_)_2_CH(CH_3_)_2_; **2-9**, -(CH_2_)_3_Cl; **2-10**, -(CH_2_)_3_Br; **2-11**, -(CH_2_)_4_Cl; **2-12**, -(CH_2_)_2_O(CH_2_)_2_O(CH_2_)_2_Cl; **2-13**, -CH_2_COC_6_H_5_; **2-14**, -(CH_2_)_2_OCH_3_; **2-15**, -(CH_2_)_2_O(CH_2_)_3_CH_3_; **2-16**, -(CH_2_)_4_OCH_3_; **2-17**, -(CH_2_)_2_O(CH_2_)_2_OCH_3_; **2-18**, -CH_2_CH(OC_2_H_5_)_2_; **2-19**, -(CH_2_)_2_OCOCH_3_; **2-20**, -(CH_2_)_3_OCOCH_3_; **2-21**, -(CH_2_)_2_OCOC_2_H_5_; **2-22**, -(CH_2_)_2_CO_2_C_2_H_5_; **2-23**, -(CH_2_)_3_CO_2_C_2_H_5_; **2-24**, (oxiran-2-yl)methyl; **2-25**, -(CH_2_)_2_OCOCH(CH_3_)_2_; **2-26**, -(CH_2_)_5_Br; **2-27**, -CH_2_CO_2_CH_2_C_2_H_5_; **2-28**, -(CH_2_)_4_CO_2_C_2_H_5_; **2-29**, -(CH_2_)_5_CO_2_C_2_H_5_. R^2^ = **3-1**, -(CH_2_)_5_-; **3-2**, -(CH_2_)_6_-; **3-3**, -(CH_2_)_2_O(CH_2_)_2_O(CH_2_)_2_-.

### 2.2. Solubility of Evodiamine Derivatives in SGF and SIF Main Text Paragraph

We aimed to improve the aqueous solubility of evodiamine, subsequently, to promote its pharmacological or pharmacokinetic peculiarity to enhance its therapeutic effects. The solubilities of N13-position evodiamine derivatives in SGF and SIF solutions were evaluated and the results are shown in Table 1. 

**Table 1:** Solubilities and cytotoxicity of N13-substituted evodiamine analogues.

**Sample**		**2-1**	**2-2**	**2-3**	**2-4**	**2-5**	**2-6**	**2-7**	**2-8**	**2-9**	**2-10**
In SGF(µg/mL)		60	>120	60–120	60	30	60	>110	>150	60–120	Nd
In SIF(µg/mL)		175	>334	322	162	72.3	142.5	>310	>373	322	Nd
IC50 *	Prostate DU-145	20	3	5	>30	12	>30	9	4	4	>30
	Prostate PC-3	10	>30	11	>30	8	25	>30	>30	9	20
	Lung NCl-H460	14	2	3	>30	8	30	12	3	5	30
	Breast MCF-7	>30	15	12	>30	10	>30	25	18	16	>30
	Colon HCT-15	19	30	17	>30	10	30	>30	>30	13	>30
	CNS SF-268	>30	3	5	>30	10	>30	11	>30	4	>30
**Sample**		**2-11**	**2-12**	**2-13**	**2-14**	**2-15**	**2-16**	**2-17**	**2-18**	**2-19**	**2-20**
In SGF(µg/mL)		98	Viscous	30–60	15–30	30–120	Viscous	60	>120	>120	>120
In SIF(µg/mL)		273	solid	73.7–147	41.6–83.1	74.4–298	solid	148	286	308	278
IC50 *	Prostate DU-145	>30	17	>30	>30	15	2.0	>30	30	15	14
	Prostate PC-3	3	9	>30	>30	10	1	>30	121	8	6
	Lung NCl-H460	9	8	>30	>30	9	1.5	>30	10	12	9
	Breast MCF-7	15	17	>30	>30	>30	1	>30	>30	15	10
	Colon HCT-15	23	19	>30	>30	14	1	>30	>30	15	13
	CNS SF-268	20	10	>30	>30	25	1.2	>30	18	10	7
**Sample**		**2-** **21**	**2-** **22**	**2-** **23**	**2-** **24**	**2-** **25**	**2-** **26**	**2-** **27**	**2-** **28**	**2-** **29**	**3-1**
In SGF(µg/mL)		>120	>120	30	20	Viscous	67.8	72.9	89.4	Viscous	>120
In SIF(µg/mL)		>308	265	71.9	55.7	solid	132.3	131.5	165.9	solid	>350
IC50 *	Prostate DU-145	17	14	>30	>30	nd	nd	nd	nd	nd	12
	Prostate PC-3	9	8	15	>30	7	9	5	7	11	9
	Lung NCl-H460	12	12	25	>30	nd	nd	nd	nd	nd	15
	Breast MCF-7	17	15	18	>30	nd	nd	nd	nd	nd	8
	Colon HCT-15	15	10	25	>30	13	15	11	30	9	15
	CNS SF-268	18	9	20	>30	21	11	17	18	8	10
**Sample**		**3-** **2**	**3-** **3**	**4-1**	**4-** **2**	**5**	**6**	**6-** **1**	**6-** **2**	**Evodiamine**	
In SGF(µg/mL)		>120	nd	>220	nd	60	60	60	60	7.5–15	
In SIF(µg/mL)		>320	nd	320	nd	154	173.9	166.7	160.4	24.8–49.5	
IC50 *	Prostate DU-145	10	nd	nd	nd	>30	>30	20	>30	2	
	Prostate PC-3	3	nd	nd	nd	>30	8	10	15	30	
	Lung NCl-H460	5	nd	25	nd	20	10	10	10	12	
	Breast MCF-7	6	nd	15	nd	30	20	15	15	>30	
	Colon HCT-15	5	nd	25	nd	>30	12	18	12	>30	
	CNS SF-268	3	nd	nd	nd	>30	15	12	15	3	

* The unit of IC_50_ is micromole/L.

It is interesting that the solubilities of these derivatives were obviously improved when compared to evodiamine. For example, the solubility of evodiamine itself was only 7.5–15 μg/mL and 24.8–49.5 µg/mL in SGF and SIF, respectively, while the solubilities in SGF and SIF for all evodiamine derivatives were nearly two fold that of evodiamine itself.

### 2.3. Activity of Evodiamine Derivatives against Human Cancer Cell Lines

To evaluate the antiproliferative effects of the evodiamine derivatives on human cancer cells, the derivatives were used to treat a variety of human cancer cell lines derived from prostate cancer (DU-145 and PC-3), lung cancer (NCl-H460), breast cancer (MCF-7), colon cancer (HCT-15) and glioblastoma (SF-268) and the growth inhibition effect was tested by MTT cytotoxicity assays using evodiamine as a reference. The results are listed in Table 1.

We observed that the appropriate substitution on N13-position of evodiamine made the derivatives more cytotoxic (Table 1, compound **2-16**, with IC_50_ less than 2 μM for all six tested cancer cell lines) and broadened antitumor spectra (Table 1, **2-2**, **2-16**, **3-2**, **2-3**, **2-5**, **2-9**, **2-20**, **3-1**, **3-2**, **6-1** and **6-2**). While improper substitution on N13-position of evodiamine, (for example, with alkyl groups, Table 1, **2-1**, **2-4**, and **2-6**) resulted in a decrease of antitumor potency. It should be noted that substitution with alkylamino-alkyl groups on the N13-position, as expected, provided us the corresponding derivatives with more solubility in water, but with only moderate antitumor potency (compounds **6-1**, **6-2**).

Moreover, we tried to make a dimer using a linker between N13-position, as shown in Table 1, which exhibited moderate (compound **3-1**) to good (compound **3-2**) antitumor activity against all the tested tumor cell lines. It should be very intriguing that the evodiamine derivative with a benzoylmethyl group (Table 1, compound **2-8**) was inactive against the six tested tumor cell lines, although it was reported that one with a benzoyl group at N13-position showed higher antitumor activity and broad spectrum activity against A549, MDA-MB-435 and HCT116 [13].

### 2.4. Analysis of Anticancer Activities by Spatial Three-Dimensional Structures

**Figure 2:**
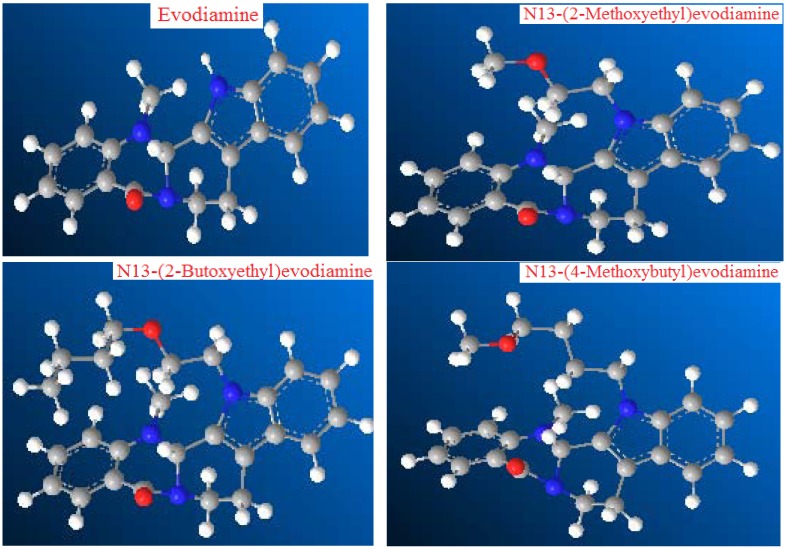
Three-dimensional models of evodiamine, N13-(2-methoxyethyl)evodiamine and N13-(2-butoxyethyl)evodiamine and N13-(4-methoxybutyl)evodiamine.

In order to elucidate the effect of the spatial structure of evodiamine derivatives on anticancer activity, three-dimensional models of evodiamine, N13-(2-methoxyethyl)evodiamine, N13-(2-butoxyethyl)evodiamine, and N13-(4-methoxybubyl)evodiamine were obtained using ChemBioDraw Ultra 11.0 [30] (Figure 2).

Comparing the three-dimensional models of these four compounds, we could not find obvious differences in respect of the spatial three-dimensional structures of the evodiamine moiety, that is, the spatial three-dimensional structure of evodiamine was not changed due to the introduction of alkoxy groups. Therefore, it could be speculated that the reason leading to the difference in the anticancer activities of these four compounds was the differences in the structures of substituents.

### 2.5. Apoptosis of Human Cancer Cell Lines Induced by Evodiamine Derivatives

The significant difference of antitumor potency resulting from such subtle change in the structures encouraged us to further explore their antitumor mechanism. Hence, compounds **2-2**, **2-3**, **2-16** and **3-2** were chosen for testing their effect on inducing apoptosis in breast MCF-7, lung NCl-H460 and colon HCT-15 cancer cell lines.

**Figure 3:**
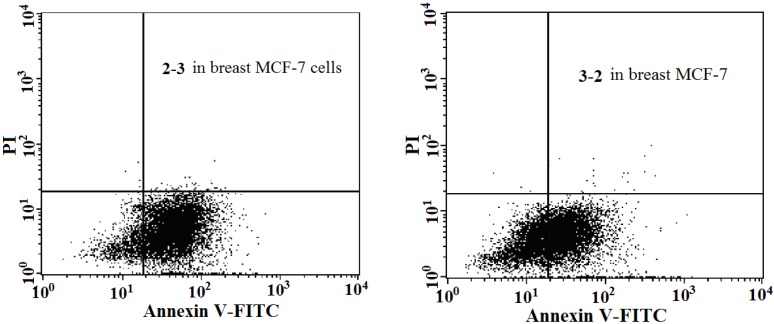
Apoposis induced by **2-3** and **3-2** in breast MCF-7 cells.

Apoptosis was determined by flow cytometry analysis using the Annexin V-FITC/PI Apoptosis Kit (Becton Dickinson, Franklin Lakes, NJ, USA) and the results are presented in Figure 3 and in Table 2. As shown in Figure 3, compounds **2-3** and **3-2** strongly induced early apoptosis in MCF-7 with the percentages increasing from 3.21% (medium control) to 84.58% and 55.62%, respectively, while **2-16** and **3-2** obviously induced late apoposis in HCT-15 with the percentages increasing from 3.55% (medium control) to 59.01% and 65.67%, respectively. These two compounds also induced late apoptosis in NCl-H460 with a moderate intensity with the percentages increasing to 40%–50%.

**Table 2:** The apoposis results against breast MCF-7, lung NCl-H460 and colon HCT-15 cancer cell lines after 48-hour treatment.

Breast MCF-7	**Treatment**	**Medium control**	**2-2**	**2-3**	**2-16**	**3-2**
	Viable cell (%)	92.58	63.35	14.70	49.09	44.07
	Early apoposis (%)	3.21	34.00	84.58	47.38	55.62
	Late apoposis (%)	5.21	2.64	0.72	3.53	0.31
Lung NCl-H460	**Treatment**	**Medium control**	**2-2**	**2-3**	**2-16**	**3-2**
	Viable cell (%)	82.63	37.82	29.22	43.75	39.89
	Early apoposis (%)	15.40	30.45	37.60	12.02	13.03
	Late apoposis (%)	1.98	31.73	33.17	44.23	47.08
Colon HCT-15	**Treatment**	**Medium control**	**2-2**	**2-3**	**2-16**	**3-2**
	Viable cell (%)	91.61	67.71	72.29	0.07	0.73
	Early apoposis (%)	4.84	16.04	13.93	40.91	33.60
	Late apoposis (%)	3.55	16.26	13.76	59.01	65.67

## 3. Experimental

### 3.1. General

Evodiamine was purchased from Aladdin Industrial Inc. (Shanghai, China). All the chemicals used were purchased from Guangzhou Chemical Reagent Company (Guangzhou, China), unless otherwise stated. Human cancer cell lines derived from prostate cancer (DU-145 and PC-3), lung cancer (NCl-H460), breast cancer (MCF-7), colon cancer (HCT-15) and glioblastoma (SF-268) were supplied by CK Life Science INT’L. Inc. (Hong Kong, China). The Annexin V-FITC/PI apoptosis Kit was purchased from Becton Dickinson (Franklin Lakes, NJ, USA).

^1^H-NMR and ^13^C-NMR spectra were recorded on a Bruker Avance 300 spectrometer (Bruker Company, Saarbrücken, Germany) using CDCl_3_ or DMSO-*d_6_* as solvent, chemical shifts were given in ppm (δ). Elemental analyses were performed with a Vario EL cube instrument (Elementar Company, Hanau, Germany). The mass spectra were recorded on a LCQ DECA XP LC-MS mass spectrometer (Thermo Company, Waltham, MA, USA) and high resolution mass spectra were recorded on a MAT95XP High Resolution Mass Spectrometer (Thermo Company). X-ray data were collected on a Smart 1000 CCD Single Crystal Diffractometer (Bruker).

### 3.2. Synthesis of N13-Substituted Evodiamine Derivatives: General Synthetic Procedure for **2-1**–**2-29**

NaH (40% oil, 7.2 mmol) was suspended in a solution of evodiamine (**1**, 1.82 g, 6.0 mmol) in DMF (30 mL). The mixture was stirred for 20 min, then 7.5 mmol of the appropriate alkylating reagent was added into the mixture, and the reaction mixture was stirred for 8 h more. The mixture was poured into H_2_O (150 mL) and then extracted with EtOAc. The extract was dried on anhydrous Na_2_SO_4_, the EtOAc was removed and the crude product was purified by column chromatography to afford the pure target compounds. The characterization data for these compounds was as follows:

*N13-Allylevodiamine* (**2-1**). Colorless solid product 1.49 g, yield 72.4%, m.p. 163–165 °C. ^1^H-NMR (CDCl_3_) δ, ppm: 2.43 (s, 3H), 2.91–2.97 (m, 1H), 3.01–3.03 (m, 1H), 3.16–3.25 (m, 1H), 4.81–4.95 (m, 3H), 4.98–5.08 (m, 2H), 5.10–5.15 (m, 1H), 5.94 (s, 1H), 7.15–7.18 (m, 1H), 7.20 (s, 1H), 7.23–7.25 (m, 1H), 7.27–7.30 (m, 1H), 7.37–7.38 (m, 1H), 7.46–7.52 (m, 1H), 7.62 (d, *J* = 7.8 Hz, 1H), 8.14 (dd, *J_1_* = 7.8 Hz, *J_2_* = 1.8 Hz, 1H). ^13^C-NMR (CDCl_3_) δ, ppm: 20.8, 36.9, 39.7, 46.5, 68.3, 110.2, 113.6, 116.9, 119.2, 120.0, 122.9, 123.3, 124.4, 126.0, 128.6, 129.2, 133.1, 133.6, 133.9, 137.5, 151.0, 164.7. ESI-MS, *m*/z 344.2 [M+H]^+^. Anal. Calcd for C_22_H_21_N_3_O: C, 76.94; H, 6.16. Found: C, 77.01; H, 6.21.

*N13-Butylevodiamine* (**2-2**). Yellowish solid product 1.25 g, yield 58.0%, m.p. 141–143 °C. ^1^H-NMR (CDCl_3_) δ, ppm: 0.96 (t, *J* = 7.5 Hz, 3H), 1.34–1.46 (m, 2H), 1.77–1.88 (m, 2H), 2.42 (s, 3H), 2.84–2.95 (m, 1H), 3.01–3.07 (m, 1H), 3.15–3.25 (m, 1H), 4.15–4.24 (m, 1H), 4.32–4.42 (m, 1H), 4.88–4.95 (m, 1H ), 5.98 (s, 1H), 7.14–7.17 (m, 1H), 7.19–7.21 (m, 1H), 7.23–7.31 (m, 2H), 7.41 (d, *J* = 8.1 Hz, 1H), 7.47–7.53 (m, 1H), 7.62 (d, *J* = 7.8 Hz, 1H), 8.15 (dd, *J*_1_ = 7.5 Hz, *J*_2 _= 1.5 Hz, 1H). ^13^C-NMR (CDCl_3_) δ, ppm: 14.3, 20.9, 32.7, 36.9, 39.7, 44.1, 68.4, 110.0, 110.1, 113.2, 119.2, 119.7, 122.7, 123.3, 124.4, 125.9, 128.6, 129.2, 133.1, 137.4, 151.1, 164.7. HRMS *m/z* (M+H)^+^: Calcd for C_23_H_26_N_3_O, 360.2070; found: 360.2084.

*N13-Pentylevodiamine* (**2-3**). Yellowish solid product 1.55 g, yield 69.3%, m.p. 143–145 °C. ^1^H-NMR (CDCl_3_) δ, ppm: 0.92 (t, *J* = 7.2 Hz, 3H), 1.37–1.39 (m, 4H), 1.83–1.87 (m, 2H), 2.43 (s, 3H), 2.85–2.96 (m, 1H), 3.02–3.07 (m, 1H), 3.16–3.26 (m, 1H), 4.14–4.24 (m, 1H), 4.31–4.41 (m, 1H), 4.90–4.95 (m, 1H), 5.98 (s, 1H), 7.14–7.22 (m, 2H), 7.24–7.27 (m, 1H), 7.29–7.31 (m, 1H), 7.39–7.41 (m, 1H), 7.48–7.54 (m, 1H), 7.63 (d, *J* = 7.8 Hz, 1H), 8.13–8.17 (m, 1H). ^13^C-NMR (CDCl_3_) δ, ppm: 14.4, 20.8, 22.8, 29.7, 30.3, 36.8, 39.7, 44.4, 68.4, 110.1, 113.2, 119.2, 119.7, 122.7, 123.3, 124.4, 125.9, 128.5, 129.2, 133.1, 137.4, 151.1, 164.7. HRMS *m/z* (M+H)^+^: Calcd for C_24_H_28_N_3_O, 374.2227 (M+H)^+^; found: 374.2238.

*N13-(3-Methyl-2-butenyl)evodiamine* (**2-4**). Yellowish viscous solid product 1.01 g, yield 45.4%. ^1^H-NMR (CDCl_3_) δ, ppm: 1.68 (d, *J* = 0.9 Hz, 3H), 1.77 (s, 3H), 2.45 (s, 3H), 2.88–2.96 (m, 1H), 3.00–3.07 (m, 1H), 3.16–3.25 (m, 1H), 4.77–4.84 (m, 1H), 4.88–4.94 (m, 1H), 5.04–5.12 (m, 1H ), 5.20–5.26 (m, 1H ), 5.93 (s, 1H), 7.14–7.17 (m, 1H), 7.19–7.20 (m, 1H), 7.22–7.25 (m, 1H), 7.27–7.30 (m, 1H), 7.35–7.38 (m, 1H), 7.46–7.51 (m, 1H), 7.61 (d, *J* = 7.5 Hz, 1H), 8.14 (dd, *J* = 7.5 Hz, 1.5 Hz, 1H). ^13^C-NMR (CDCl_3_) δ, ppm: 18.4, 20.8, 25.9, 36.9, 39.7, 42.3, 68.3, 110.0, 113.3, 119.2, 119.7, 120.7, 122.7, 123.2, 124.3, 126.0, 128.5, 129.1, 133.0, 135.0, 137.4, 151.0, 164.8. HRMS *m/z* (M+H)^+^: Calcd for C_24_H_26_N_3_O, 372.2070; found: 372.2068.

*N13-Octylevodiamine* (**2-5**). Yellowish solid product 1.77 g, yield 71.0%, m.p. 65–67 °C. ^1^H-NMR (CDCl_3_) δ, ppm: 0.88 (t, *J* = 7.5 Hz, 3H), 1.26–1.30 (m, 8H), 1.35–1.39 (m, 2H), 1.78–1.82 (m, 2H), 2.38 (s, 3H), 2.88–2.93 (m, 1H), 2.97–3.05 (m, 1H), 3.11–3.24 (m, 1H), 4.11–4.17 (m, 1H), 4.29–4.39 (m, 1H), 4.87–4.93 (m, 1H), 5.92 (s, 1H), 7.11–7.14 (m, 1H), 7.15–7.18 (m, 1H), 7.21–7.22 (m, 1H), 7.24–7.26 (m, 1H), 7.29–7.33 (m, 1H), 7.43–7.50 (m, 1H), 7.60 (d, *J* = 7.5 Hz, 1H), 8.15 (d, *J* = 7.8 Hz, 1H). ^13^C-NMR (CDCl_3_) δ, ppm: 14.5, 20.8, 22.9, 23.0, 27.5, 29.6, 30.5, 32.1, 36.8, 39.7, 44.3, 68.4, 110.0, 113.2, 119.2, 119.7, 122.7, 123.2, 124.3, 125.9, 128.5, 129.2, 133.0, 137.3, 151.1, 164.7. HRMS *m/z* (M+H)^+^: Calcd for C_27_H_34_N_3_O, 416.2696; found: 416.2696.

*N13-(3-Phenylpropyl)evodiamine* (**2-6**). Colorless solid product 1.52 g, yield 60.1%, m.p. 158–160 °C. ^1^H-NMR (CDCl_3_) δ, ppm: 2.23–2.25 (m, 2H), 2.38 (s, 3H), 2.69 (t, *J* = 7.2 Hz, 2H), 2.86–3.06 (m, 1H), 3.13–3.22 (m, 2H), 4.20–4.25 (m, 1H), 4.32–4.42 (m, 1H), 4.86–4.93 (m, 1H), 5.84 (s, 1H), 7.09–7.12 (m, 1H), 7.13–7.16 (m, 3H), 7.18–7.20 (m, 1H), 7.21–7.25 (m, 3H), 7.28–7.31 (m, 2H), 7.46–7.52 (m, 1H), 7.61 (d, *J* = 8.7 Hz,1H), 8.14 (dd, *J* = 8.7 Hz, 1.2 Hz, 1H). ESI-MS, *m*/*z* 422.2 [M+H]^+^. Anal. Calcd. for C_28_H_27_N_3_O: C, 79.78; H, 6.46. Found: C, 79.66; H, 6.54. 

*N13-Isobutylevodiamine* (**2-7**). Yellowish solid product 1.18 g, yield 54.8%, m.p. 154–156 °C. ^1^H-NMR (CDCl_3_) δ, ppm: 0.86 (d, *J* = 6.6 Hz, 3H), 0.98 (d, *J* = 6.6 Hz, 3H), 2.23–2.32(m, 1H), 2.39 (s, 3H), 2.84–2.95 (m, 1H), 3.01–3.08 (m, 1H), 3.16–3.26 (m, 1H), 4.04–4.11 (m, 1H), 4.16–4.23 (m, 1H), 4.89–4.95 (m, 1H ), 5.99 (s, 1H), 7.13–7.20 (m, 2H), 7.23–7.29 (m, 2H), 7.37–7.40 (m, 1H), 7.47–7.52 (m, 1H), 7.61 (d, *J* = 7.5 Hz, 1H), 8.13–8.16 (m, 1H). ^13^C-NMR (CDCl_3_) δ, ppm: 20.8, 20.9, 29.8, 30.1, 36.7, 39.6, 39.7, 51.7, 68.5, 110.0, 110.5, 113.4, 119.1, 119.7, 122.7, 123.1, 124.2, 125.8, 128.9, 129.2, 133.1, 137.8, 151.1, 164.7. ESI-MS, *m*/*z* 360.2 [M+H]^+^. Anal. Calcd. for C_23_H_25_N_3_O: C, 76.85; H, 7.01. Found: C, 76.76; H, 7.14. 360.2070.

*N13-Isopentylevodiamine* (**2-8**). Yellowish solid product 1.10 g, yield 49.2%, mp 108–110 °C. ^1^H-NMR (CDCl_3_) δ, ppm: 1.00 (d, *J* = 6.6 Hz, 3H), 1.03 (d, *J* = 6.3 Hz, 3H), 1. 67–1.78 (m, 3), 2.42 (s, 3H), 2.89–2.96 (m, 1H), 3.01–3.07 (m, 1H), 3.15–3.25 (m, 1H), 4.14–4.23 (m, 1H), 4.33–4.43 (m, 1H), 4.89–4.95 (m, 1H), 5.97 (s, 1H), 7.14–7.17 (m, 1H), 7.19–7.20 (m, 1H), 7.22–7.26 (m, 1H), 7.27–7.31 (m, 1H), 7.37–7.40 (m, 1H), 7.47–7.53 (m, 1H), 7.62 (d, *J* = 7.5 Hz, 1H), 8.16 (dd, *J*_1_ = 7.8 Hz, *J*_2_ =1.5 Hz, 1H). ^13^C-NMR (CDCl_3_) δ, ppm: 20.8, 22.9, 23.1, 26.8, 36.9, 39.3, 39.7, 42.8, 68.5, 109.9, 113.1, 119.2, 119.7, 122.7, 123.3, 124.5, 125.9, 128.4, 129.2, 133.1, 137.3, 151.1, 164.7. HRMS *m/z* (M+H)^+^: Calcd for C_24_H_28_N_3_O, 374.2227; found: 374.2227.

*N13-(3-Chloropropyl)evodiamine* (**2-9**). Yellowish solid product 0.96 g, yield 42.1%, m.p. 118–120 °C. ^1^H-NMR (CDCl_3_) δ, ppm: 2.27–2.38 (m, 2H), 2.40 (s, 3H), 2.85–2.96 (m, 1H), 3.01–3.07 (m, 1H), 3.21 (td, *J*_1_ = 12.6 Hz, *J*_2_ = 3.9 Hz, 1H), 3.51–3.65 (m, 2H), 4.33–4.43 (m, 1H), 4.56–4.65 (m, 1H), 4.89–4.96 (m, 1H), 6.01 (s, 1H), 7.16–7.22 (m, 2H), 7.24–7.33 (m, 2H), 7.43–7.53 (m, 2H), 7.62 (d, *J* = 7.8 Hz, 1H), 8.15 (dd, *J*_1_ = 7.8 Hz, *J*_2_ = 1.5 Hz, 1H). ^13^C-NMR (CDCl_3_) δ, ppm: 20.7, 33.1, 36.8, 39.6, 41.4, 42.5, 68.4, 109.7, 113.8, 119.3, 120.1, 123.1, 123.5, 124.4, 126.0, 128.6, 129.1, 133.1, 137.5, 151.0, 164.7. HRMS *m/z* (M+H)^+^: Calcd for C_22_H_23_ClN_3_O, 380.1524; found 380.1527.

*N13-(3-Bromopropyl)evodiamine* (**2-10**). Colorless solid product 1.41 g, yield 55.4%, m.p. 130–132 °C. ^1^H-NMR (CDCl_3_) δ, ppm: 2.36–2.48 (s, 5H), 2.86–2.96 (m, 1H), 3.01–3.07 (m, 1H), 3.15–3.25 (m, 1H), 3.37–3.47 (m, 2H), 4.32–4.42 (m, 1H), 4.55–4.65 (m, 1H), 4.89–4.95 (m, 1H ), 5.94 (s, 0.16 H), 6.02 (s, 0.84H), 7.16–7.20 (m, 1H), 7.21–7.22 (m, 1H), 7.24–7.28 (m, 1H), 7.30–7.36 (m, 1H), 7.44–7.53 (m, 2H), 7.62 (d, *J* = 7.8 Hz, 1H), 8.15 (dd, *J*_1_ = 7.8 Hz, *J*_2_ =1.5 Hz, 1H). ^13^C-NMR (CDCl_3_) δ, ppm: 20.8, 30.8, 33.3, 36.9, 39.6, 42.6, 68.4, 109.9, 113.8, 119.3, 120.1, 123.1, 123.5, 124.4, 124.6, 126.0, 128.6, 129.2, 133.1, 137.5, 151.0, 164.6. HRMS *m/z* (M+H)^+^: Calcd for C_22_H_23_BrN_3_O, 424.1019; found: 424.1015.

*N13-(4-Chlorobutyl)evodiamine* (**2-11**). Colorless solid product 1.17 g, yield 49.5%, m.p. 127–129 °C. ^1^H-NMR (CDCl_3_) δ, ppm: 1.82–1.91 (m, 2H), 2.00–2.10 (m, 2H), 2.42 (s, 3H), 2.85–2.96 (m, 1H), 3.01–3.07 (m, 1H), 3.15–3.25 (m, 1H), 3.50–3.62 (m, 2H), 4.09–4.27 (m, 1H), 4.36–4.46 (m, 1H), 4.89–4.95 (dd, *J*_1_ = 12.6 Hz, *J*_2_ = 4.8 Hz, 1H), 5.97 (s, 1H), 7.15–7.25 (m, 3H), 7.27–7.32 (m, 1H), 7.38–7.41 (m, 1H), 7.51 (t, *J* = 7.8 Hz, 1H), 7.62 (d, *J* = 7.8 Hz, 1H), 8.15 (d, *J* = 7.8 Hz, 1H). ^13^C-NMR (CDCl_3_) δ, ppm: 20.8, 30.8, 33.3, 36.9, 39.6, 42.6, 68.4, 109.9, 113.8, 119.3, 120.1, 123.1, 123.5, 124.4, 124.6, 126.0, 128.6, 129.2, 133.1, 137.5, 151.0, 164.6. HRMS *m/z* (M+H)^+^: Calcd for C_23_H_25_ClN_3_O, 394.1681; found: 394.1677.

*N13-(2-(2-(2-Chloroethoxy)ethoxy)ethyl)evodiamine* (**2-12**). Colorless solid product 0.87 g, yield 31.3%, m.p. 126–128 °C. ^1^H-NMR (CDCl_3_) δ, ppm: 2.34 (s, 3H), 2.82–2.93 (m, 2H), 3.07–3.15 (m, 1H), 3.35–3.37 (m, 3H), 3.39–3.41 (m, 5H), 3.71–3.73 (m, 2H), 4.27–4.33 (m, 1H), 4.63–4.71 (m, 1H ), 4.81–4.87 (m, 1H ), 6.07 (s, 1H), 7.11–7.21 (m, 4H), 7.37 (d, *J* = 8.1 Hz, 1H), 7.40–7.43 (m, 1H), 7.55 (d, *J* = 7.8 Hz, 1H), 8.08 (dd, *J*_1_ = 7.8 Hz, *J*_2_ = 1.2 Hz, 1H). ^13^C-NMR (CDCl_3_) δ, ppm: 20.4, 35.5, 39.3, 42.7, 43.8, 67.8, 70.1, 70.6, 70.7, 71.3, 109.8, 113.2, 119.0, 120.1, 122.6, 123.1, 124.2, 124.4, 126.1, 128.9, 129.4, 132.9, 137.2, 151.8, 163.9. HRMS *m/z* (M+H)^+^: Calcd for C_25_H_29_ClN_3_O_3_, 454.1892; found: 454.1905.

*N13-(Benzonylmethyl)evodiamine* (**2-13**). Yellowish solid product 0.53 g, yield 21.0%, m.p. 245 °C (decomposed). ^1^H-NMR (CDCl_3_) δ, ppm: 2.35 (s, 3H), 2.94–3.00 (m, 1H), 3.06–3.11 (m, 1H), 3.22–3.31 (m, 1H), 4.89–4.95 (m, 1H), 561 (s, 0.23H), 5.67 (s, 1.61H), 5.74 (s, 0.17H), 5.87 (s, 1H), 6.73–6.76 (m, 1H), 7.17–7.24 (m, 3H), 7.27–7.37 (m, 2H), 7.52–7.57 (m, 2H), 7.63–7.70 (m, 2H), 8.07–8.15 (m, 3H). ESI-MS, *m*/*e* 422.2 [M+H]^+^. Anal. Calcd for C_27_H_23_N_3_O_2_: C, 76.94; H, 5.50. Found: C, 76.96; H, 5.54.

*N13-(2-Methoxyethyl)evodiamine* (**2-14**). Colorless solid product 1.31 g, yield 60.5%, m.p. 166–168 °C. ^1^H-NMR (CDCl_3_) δ, ppm: 2.37 (s, 3H), 2.73–2.83 (m, 1H), 2.95–2.08 (m, 2H), 3.15 (s, 3H), 3.62 (t, *J* = 8.1, Hz 2H), 4.40–4.57 (m, 2H), 4.62–4.68 (m, 1H), 6.13 (s, 1H), 7.07 (t, *J* = 7.5 Hz, 1H), 7.17–7.23 (m, 2H), 7. 31 (d, *J* = 7.8 Hz, 1H), 7.50–7.57 (m, 3H), 7.92 (d, *J* = 7.8 Hz, 1H). ESI-MS, *m*/*e* 362.2 [M+H]^+^. Anal. Calcd for C_22_H_23_N_3_O_2_: C, 73.11; H, 6.41. Found: C, 73.06; H, 6.54.

*N13-(2-Butoxyethyl)evodiamine* (**2-15**). Colorless solid product 1.42 g, yield 58.7%, m.p. 68–70 °C. ^1^H-NMR (CDCl_3_) δ, ppm: 0.78 (t, *J* = 7.2 Hz, 3H), 1.14–1.22 (m, 2H), 1.34–1.41 (m, 2H), 2.41 (s, 3H), 2.84–2.94 (m, 1H), 3.00–3.06 (m, 1H), 3.14–3.20 (m, 1H), 3.22–3.30 (m, 1H), 3.32–3.38 (m, 1H), 3.68–3.71 (m, 2H), 4.30–4.38 (m, 1H), 4.65–4.75 (m, 1H), 4.88–4.94 (m, 1H), 6.11 (s, 1H), 7.14–7.17 (m, 1H), 7.19–7.20 (m, 1H), 7.22–7.30 (m, 2H), 7.40–7.43 (m, 1H), 7.46–7.52 (m, 1H), 7.61 (dd, *J*_1_ = 7.8 Hz, *J*_2_ = 0.9 Hz, 1H), 8.14 (dd, *J*_1_ = 7.8 Hz, *J*_2_ = 1.2 Hz, 1H). ^13^C-NMR (CDCl_3_) δ, ppm: 14.2, 19.5, 20.8, 32.0, 36.8, 39.6, 44.2, 68.3, 69.7, 71.5, 110.0, 113.3, 119.1, 119.8, 122.7, 123.2, 124.2, 124.3, 126.1, 129.1, 129.3, 133.0, 137.4, 151.1, 164.7. HRMS *m/z* (M+H)^+^: Calcd for C_25_H_30_N_3_O_2_, 404.2333; found: 404.2341.

*N13-(4-Methoxybutyl)evodiamine* (**2-16**). Yellowish viscous solid product 1.28 g, yield 54.8%. ^1^H-NMR (CDCl_3_) δ, ppm: 1.58–1.68 (m, 2H), 1.86–1.96 (m, 2H), 2.41 (s, 3H), 2.84–2.94 (m, 1H), 3.00–3.08 (m, 1H), 3.15–3.23 (m, 1H), 3.25 (s, 3H), 3.37 (t, *J* = 6.3 Hz, 2H), 4.17–4.48 (m, 1H), 4.38–4.48 (m, 1H), 4.88–4.94 (m, 1H), 5.98 (s, 1H), 7.14–7.18 (m, 1H), 7.18–7.21 (m, 1H), 7.23–7.30 (m, 2H), 7.38–7.41 (m, 1H), 7.46–7.52 (m, 1H), 7.61 (d, *J* = 7.8 Hz, 1H), 8.15 (dd, *J*_1_ = 7.8 Hz, *J*_2_ = 1.2 Hz, 1H). ^13^C-NMR (CDCl_3_) δ, ppm: 20.8, 27.5, 27.6, 36.8, 39.7, 44.1, 58.9, 68.4, 72.5, 110.0, 113.3, 119.2, 119.7, 122.8, 123.3, 124.2, 124.3, 125.9, 128.5, 129.1, 133.0, 137.3, 151.1, 164.7. ESI-Ms, *m*/*e* 404.2 [M+H]^+^. Anal. Calcd for C_24_H_27_N_3_O_2_: C, 74.01; H, 6.99. Found: C, 73.96; H, 6.94. 

*N13-(2-(2-Methoxyethoxy)ethyl)evodiamine* (**2-17**). Colorless solid product 1.73 g, yield 71.2%, m.p. 116–118 °C. ^1^H-NMR (CDCl_3_) δ, ppm: 2.41 (s, 3H), 2.83–2.94 (m, 1H), 2.98–3.06 (m, 1H), 3.14–3.21 (m, 1H), 3.23 (s, 3H), 3.35–3.28 (m, 2H), 3.40–3.44 (m, 1H), 3.45–3.52 (m, 1H), 3.71–3.84 (m, 2H), 4.32–4.40 (m, 1H), 4.68–4.78 (m, 1H), 4.88–4.94 (m, 1H), 6.13 (s, 1H), 7.14–7.17 (m, 1H), 7.19 (s, 1H), 7.22–7.26 (m, 1H), 7.27–7.30 (m, 1H), 7.43 (d, *J* = 8.1 Hz, 1H), 7.49 (td, *J*_1_ = 7.5 Hz, *J*_2_ = 1.5 Hz, 1H), 7.61 (d, *J* = 7.8 Hz, 1H), 8.14 (dd, *J*_1_ = 7.8 Hz, *J*_2_ = 1.8 Hz, 1H). ^13^C-NMR (CDCl_3_) δ, ppm: 20.8, 36.8, 39.6, 44.1, 59.3, 68.3, 70.2, 71.0, 72.2, 110.0, 113.4, 119.2, 119.9, 122.8, 123.3, 124.3, 126.1, 128.5, 129.1, 129.3, 133.0, 137.4, 151.1, 164.8. HRMS *m/z* (M+H)^+^: Calcd for C_24_H_28_N_3_O_3_, 406.2125; found: 406.2137.

*N13-(2,2-Diethoxyethyl)evodiamine* (**2-18**). Yellowish solid product 0.43 g, yield 18.1%, m.p. 117–119 °C. ^1^H-NMR (CDCl_3_) δ, ppm: 0.83 (t, *J* = 6.9 Hz, 3H), 1.03 (t, *J* = 6.9 Hz, 3H), 2.35 (s, 3H), 2.72–2.83 (m, 1H), 2.96–3.14 (m, 3H), 3.41–3.52 (m, 2H), 3.59–3.64 (m, 1H), 4.34–4.37 (m, 2H), 4.64–4.73 (m, 2H), 6.18 (s, 1H), 7.05–7.10 (m, 1H), 7.17–7.24 (m, 2H), 7.32 (d, *J* = 7.8 Hz, 1H), 7.51–7.57 (m, 3H), 7.92 (d, *J* = 7.8 Hz, 1H). HRMS *m/z* (M+H)^+^: Calcd for C_25_H_30_N_3_O_3_, 420.2282; found 420.2298.

*N13-(2-Acetoxyethyl)evodiamine* (**2-19**). Yellowish product 1.22 g, yield 53.8%, m.p. 211–213 °C. ^1^H-NMR (CDCl_3_) δ, ppm: 2.03 (s, 3H), 2.39 (s, 3H), 2.73–2.83 (m, 1H), 2.95–3.08 (m, 1H), 3.64–3.69 (m, 2H), 4.30–4.43 (m, 2H), 4.62–4.70 (m, 1H), 4.88–4.92 (m, 1H), 6.20 (s, 1H), 7.11 (d, *J* = 7.5 Hz, 1H), 7.19–7.25 (m, 2H), 7.33 (d, *J* = 7.5 Hz, 1H), 7.52–7.60 (m, 3H), 7.94 (d, *J* = 7.8 Hz, 1H). ESI-MS, *m*/*z* 390.2 [M+H]^+^. Anal. Calcd for C_23_H_23_N_3_O_3_: C, 70.93; H, 5.95. Found: C, 71.06; H, 6.04.

*N13-(3-Acetoxypropyl)evodiamine* (**2-20**). Yellowish viscous product 1.33 g, yield 56.6%. ^1^H-NMR (CDCl_3_) δ, ppm: 1.91 (s, 3H), 2.18–2.22 (m, 2H), 2.42 (s, 3H), 2.91–2.96 (m, 1H), 3.03–3.08 (m, 1H), 3.16–3.26 (m, 1H), 4.04–4.10 (m, 1H), 4.15–4.21 (m, 1H), 4.27–4.32 (m, 1H), 4.53–4.58 (m, 1H), 4.90–4.96 (m, 1H), 5.99 (s, 1H), 7.20–7.34 (m, 4H), 7.40–7.43 (m, 1H), 7.50–7.52 (m, 1H), 7.65 (d, *J* = 7.8 Hz, 1H), 8.17 (d, *J* = 7.8 Hz, 1H). ^13^C-NMR (CDCl_3_) δ, ppm: 20.1, 20.7, 29.2, 36.4, 39.3, 40.6, 61.7, 68.0, 109.5, 113.4, 119.0, 119.8, 122.8, 123.2, 124.1, 124.4, 125.8, 128.3, 129.0, 132.9, 137.1, 150.9, 164.6, 170.9. HRMS *m/z* (M+H)^+^: Calcd for C_24_H_26_N_3_O_3_; 404.1969; found: 404.1966.

*N13-(2-Propionyloxyethyl)evodiamine* (**2-21**). Colorless solid product 1.01 g, yield 43.0%, m.p. 205–206 °C. ^1^H-NMR (CDCl_3_) δ, ppm: 1.02 (t, *J* = 7.5 Hz, 3H), 2.21 (q, *J* = 7.5 Hz, 2H), 2.41 (s, 3H), 2.84–3.07 (m, 2H), 3.21 (td, *J*_1_ = 12.6 Hz, *J*_2_ = 3.9 Hz, 1H), 4.38–4.47 (m, 3H), 4.68–4.79 (m, 1H), 4.89–4.96 (m, 1H), 6.01 (s, 1H), 7.16–7.20 (m, 1H), 7.21–7.23 (m, 1H), 7.25–7.27 (m, 1H), 7.30–7.32 (m, 1H), 7.43–7.46 (m, 1H), 7.48–7.53 (m, 1H), 7.61 (d, *J* = 7.8 Hz, 1H), 8.14 (dd, *J*_1_ = 8.1 Hz, *J*_2_ = 1.5 Hz, 1H). HRMS *m/z* (M+H)^+^: Calcd for C_24_H_26_N_3_O_3_, 404.1969; found: 404.1979.

*N13-(2-(Ethoxycarbonyl)ethyl)evodiamine* (**2-22**). Colorless viscous solid product 1.36 g, yield 57.9%. ^1^H-NMR (CDCl_3_) δ, ppm: 1.21 (t, *J* = 7.2 Hz, 3H), 2.42 (s, 3H), 2.89–2.90 (m, 3H), 3.01–3.07 (m, 1H), 3.16–3.21 (m, 1H), 4.10 (q, *J* = 7.2 Hz, 2H), 4.43–4.53 (m, 1H), 4.73–4.83 (m, 1H), 4.91–4.97 (m, 1H), 6.09 (s, 1H), 7.21–7.33 (m, 4H), 7.46 (d, *J* = 8.4 Hz, 1H), 7.50–7.53 (m, 1H), 7.64 (d, *J* = 7.8 Hz, 1H), 8.18 (d, *J* = 7.8 Hz, 1H). ^13^C-NMR (CDCl_3_) δ, ppm: 14.1, 20.4, 34.7, 36.6, 39.3, 39.5, 60.9, 68.0, 109.6, 113.7, 119.1, 119.9, 122.9, 123.3, 124.2, 124.5, 125.9, 128.3, 129.0, 133.0, 136.9, 150.7, 164.5, 171.2. HRMS *m/z* (M+H)^+^: Calcd for C_24_H_26_N_3_O_3_, 404.1969; found 404.1969.

*N13-(3-(Ethoxycarbonyl)propyl)evodiamine* (**2-23**). Colorless solid product 1.40 g, yield 57.5%, m.p. 265–268 °C. ^1^H-NMR (CDCl_3_) δ, ppm: 1.20 (t, *J* = 7.2 Hz, 3H), 2.13–2.22 (m, 2H), 2.35–2.43 (m, 5H), 2.88–3.07 (m, 2H), 3.22 (td, *J*_1_ = 12.6 Hz, *J*_2_ = 3.9 Hz, 1H), 3.99–4.12 (m, 2H), 4.21–4.30 (m, 1H), 4.41–4.51 (m, 1H), 4.89–4.95 (m, 1H), 5.97 (s, 1H), 7.15–7.19 (m, 1H), 7.20–7.21 (m, 1H), 7.23–7.27 (m, 1H), 7.30–7.32 (m, 1H), 7.43–7.52 (m, 2H), 7.62 (d, *J* = 7.8 Hz, 1H), 8.15 (dd, *J*_1_ = 7.8 Hz, *J*_2_ = 1.5 Hz, 1H). ^13^C-NMR (CDCl_3_) δ, ppm: 14.5, 20.7, 25.6, 31.8, 36.8, 39.6, 43.3, 60.9, 68.3, 110.0, 113.6, 119.2, 119.9, 123.0, 123.3, 124.4, 125.9, 128.4, 129.1, 133.1, 137.4, 151.0, 164.7, 172.7. HRMS *m/z* (M+H)^+^: Calcd for C_25_H_28_N_3_O_3_, 418.2125; found: 418.2123.

*N13-((Oxiran-2-yl)methyl)evodiamine* (**2-24**). Yellowish solid product 0.63 g, yield 30.6%, m.p. 189–191 °C.^1^H-NMR (CDCl_3_) δ, ppm: 2.21–2.23 (m, 1H), 2.39 (s, 3H), 2.69 (t, *J* = 4.2 Hz, 1H), 2.83–2.93 (m, 1H), 2.99–3.05 (m, 1H), 3.16–3.21 (m, 1H), 3.23–3.27 (m, 1H), 4.43 (d, *J* = 3.6 Hz, 0.5 H), 4.48 (d, *J* = 3.6 Hz, 0.5H), 4.87–4.90 (m, 1H), 4.94 (d, *J* = 3.3 Hz, 1H), 6.06 (s, 1H), 7.16–7.21 (m, 2H), 7.23–7.27 (m, 1H), 7.28–7.34 (m, 1H), 7.40–7.43 (m, 1H), 7.47–7.52 (m, 1H), 7.58–7.62 (m, 1H), 8.13 (dd, *J*_1_ = 7.8 Hz, *J*_2_ = 1.5 Hz, 1H). HRMS *m/z* (M+H)^+^: Calcd for C_2__2_H_2__2_N_3_O_2_, 360.1707; found: 360.1712.

*N13-(2-iso-Butyryloxyethyl)evodiamine* (**2-25**). Viscous solid product 1.42 g, yield 56.7%. ^1^H-NMR (CDCl_3_) δ, ppm: 1.01–1.05 (m, 6H), 2.36–2.46 (m, 4H), 2.84–2.95 (m, 1H), 3.01–3.07 (m, 1H), 3.16–3.25 (m, 1H), 4.38–4.48 (m, 3H), 4.69–4.80 (m, 1H), 4.89–4.96 (m, 1H), 6.00 (s, 1H), 7.16–7.20 (m, 1H), 7.21–7.23 (m, 1H), 7.25–7.28 (m, 2H), 7.44–7.47 (m, 1H), 7.48–7.53 (m, 1H), 7.59–761 (m, 1H), 8.14 (dd, *J*_1_ = 7.8 Hz, *J*_2_ = 1.2 Hz, 1H). ^13^C-NMR (CDCl_3_) δ, ppm: 19.1, 20.7, 34.2, 36.9, 39.6, 42.8, 63.2, 68.4, 110.0, 114.0, 119.2, 120.1, 123.1, 123.5, 124.4, 124.6, 126.0, 128.6, 129.2, 133.1, 137.7, 151.0, 164.6, 176.8. HRMS *m/z* (M+H)^+^: Calcd for C_25_H_28_N_3_O_3_, 418.2125; found: 418.2122.

*N13-(5-Bromopentyl)evodiamine* (**2-26**). Yellowish solid product 1.74 g, yield 64.1%. ^1^H-NMR (CDCl_3_) δ, ppm: 1.52–1.62 (m, 2H), 1.86–1.93 (m, 4H), 2.42 (s, 3H), 2.84–2.95 (m, 1H), 3.01–3.07 (m, 1H), 3.15–3.25 (m, 1H), 3.37–3.45 (m, 2H), 4.15–4.25 (m, 1H), 4.33–4.43 (m, 1H), 4.89–4.95 (m, 1H), 5.97 (s, 1H), 7.15–7.22 (m, 2H), 7.24–7.32 (m, 2H), 7.37–7.39 (m, 1H), 7.48–7.53 (m, 1H), 7.59–7.62 (m, 1H), 8.16 (dd, *J* = 7.8 Hz, 1.2 Hz, 1H). ^13^C-NMR (CDCl_3_) δ, ppm: 20.8, 26.1, 29.7, 32.2, 32.7, 33.7, 39.7, 44.1, 68.4, 109.9, 113.4, 119.3, 119.8, 122.9, 123.3, 124.4, 124.5, 126.0, 128.5, 129.2, 133.1, 137.3, 151.0, 164.7. HRMS *m/z* (M+H)^+^: Calcd for C_24_H_27_BrN_3_O, 452.1332; found: 452.1343.

*N13-(Propoxycarbonylmethyl)evodiamine* (**2-27**). Yellowish solid product 1.65 g, yield 68.2%, m.p. 168–170 °C. ^1^H-NMR (CDCl_3_) δ, ppm: 0.88 (t, J = 7.5 Hz, 3H), 1.58–1.70 (m, 2H), 2.43 (s, 3H), 2.94–2.99 (m, 1H), 3.09 (d, *J* = 13.8 Hz, 1H), 3.21–3.31 (m, 1H), 4.07–4.18 (m, 2H), 4.94–5.00 (m, 2H), 5.23 (d, *J* = 8.7 Hz, 1H ), 5.96 (s, 1H), 7.18–7.22 (m, 2H), 7.28–7.32 (m, 3H), 7.49–7.55 (m, 1H), 7.65 (d, J = 7.5 Hz, 1H), 8.17 (dd, *J_1_* = 7.8 Hz, *J_2_* = 1.5 Hz,1H). ^13^C-NMR (CDCl_3_) δ, ppm: 10.3, 20.2, 21.9, 36.8, 39.3, 45.3, 67.2, 68.0, 109.1, 114.0, 119.2, 120.2, 123.2, 123.5, 124.3, 124.6, 126.0, 128.4, 129.0, 132.9, 137.8, 150.8, 164.5, 171.7. HRMS *m/z* (M+H)^+^: Calcd for C_24_H_26_N_3_O_3_, 404.1969; found: 404.1985.

*N13-(4-(Ethoxycarbonyl)butyl)evodiamine* (**2-28**). Solid product 1.89 g, yield 70.7%, m.p. 251–253 °C. ^1^H-NMR (CDCl_3_) δ, ppm: 1.14 (t, *J* = 7.2 Hz, 3H), 1.30–1.38 (m, 2H), 1.54–1.61 (m, 2H), 1.78 (t, *J* = 7.8 Hz, 2H), 2.32 (s, 3H), 2.77–2.81 (m, 1H), 2.85–2.93 (m, 1), 3.06–3.12 (m, 1H), 4.03 (q, *J* = 7.2 Hz, 2H), 4.23–4.33 (m, 1H), 4.73–4.83 (m, 1H ), 4.91–4.97 (m, 1H ), 6.09 (s, 1H), 7.21–7.33 (m, 4H), 7.46 (d, *J* = 8.4 Hz, 1H), 7.50–7.53 (m, 1H), 7.64 (d, *J* = 7.8 Hz, 1H), 8.18 (d, *J* = 7.8 Hz, 1H). ^13^C-NMR (CDCl_3_) δ, ppm: 14.1, 20.4, 34.7, 36.6, 39.3, 39.5, 60.9, 68.0, 109.6, 113.7, 119.1, 119.9, 122.9, 123.3, 124.2, 124.5, 125.9, 128.3, 129.0, 133.0, 136.9, 151.7, 164.5, 171.2. HRMS *m/z* (M+H)^+^: Calcd for C_2__6_H_3__0_N_3_O_3_, 432.2238; found: 432.2216.

*N13-(5-(Ethoxycarbonyl)pentyl)evodiamine* (**2-29**). Viscous solid product 1.59 g, yield 57.7%. ^1^H-NMR (CDCl_3_) δ, ppm: 1.21–1.28 (m, 5H), 1.71–1.75 (m, 2H), 1.88–1.1.93 (m, 2H), 2.35 (t, *J* = 7.5 Hz, 2H), 2.41 (s, 3H), 2.91–2.96 (m, 1H), 3.03 (d, *J* = 3.3 Hz, 1H), 3.07–3.22(m, 1H), 4.12 (q, *J* = 7.2 Hz, 2H), 4.19–4.27 (m, 1H), 4.36–4.43 (m, 1H), 4.90–4.97 (m, 1H ), 5.99 (s, 1H), 7.19–7.31 (m, 4H), 7.42 (d, *J* = 8.1 Hz, 1H), 7.50–7.53 (m, 1H), 7.64 (d, *J* = 7.8 Hz, 1H), 8.18 (dd, *J_1_* = 8.1 Hz, *J_2_* = 1.5 Hz, 1H). HRMS *m/z* (M+H)^+^: Calcd for C_2__7_H_3__2_N_3_O_3_, 446.2438; found 446.2440.

### 3.3. Synthetic Procedure for **3-1–3-3**

NaH (40% oil, 7.2 mmol) was suspended in a solution of evodiamine (**1**, 1.82 g, 6.0 mmol) in DMF (30 mL). The mixture was stirred for 20 min, 2.6 mmol of the appropriate alkylating reagent was added into the mixture, and the rest of the protocol was as described above.

*1,5-Bis-(evodiamin-N13-yl)pentane* (**3-1**). Solid product 0.67 g, yield 38.3%, m.p. 217–219 °C. ^1^H-NMR (CDCl_3_) δ, ppm: 1.83 (s, 2H), 1.94–1.99 (m, 4H), 2.20–2.23 (m, 6H), 2.88–3.17 (m, 6H), 4.13–4.16 (m, 2H), 4.31–4.39 (m, 2H), 4.82–4.88 (m, 2H), 5.76 (s, 1.2H), 5.86 (s, 0.8H), 6.69 (s, 1.16H), 6.96 (s, 0.84), 7.18–7.29 (m, 6H), 7.31–7.46 (m, 4H), 7.64 (d, *J* = 8.1 Hz, 2H), 8.13 (dd, *J*_1_ = 7.8 Hz, *J*_2_ = 1.5 Hz, 2H). ^13^C-NMR (CDCl_3_) δ, ppm: 20.3, 27.5, 28.3, 36.4, 39.3, 43.6, 68.0, 109.7, 113.4, 119.2, 119.8, 122.8, 123.0, 124.1, 124.4, 125.7, 128.3, 129.0, 133.0, 137.3, 150.7, 164.4. ESI-MS, *m*/*e* 675.3 [M+H]^+^. Anal. Calcd for C_43_H_42_N_6_O_2_: C, 76.53; H, 6.27. Found: C, 76.56; H, 6.34.

*N1,6-Bis-(evodiamin-N13-yl)hexane* (**3-2**). Colorless solid product 0.96 g, yield 53.6%, m.p. 131–133 °C. ^1^H-NMR (CDCl_3_) δ, ppm: 1.35 (s, 4H), 1.78 (s, 4H), 2.36 (s, 6H), 2.82–2.91 (m, 2H), 2.99–3.03 (m, 2H), 3.10–3.21(m, 2H), 4.08–4.17 (m, 2H), 4.28–4.38 (m, 2H), 4.91 (dd, *J*_1_ = 12.6 Hz, *J*_2_ = 3.3 Hz, 2H), 5.92 (s, 2H), 7.12–7.18 (m, 4H), 7.20–7.31 (m, 6H), 7.44–7.49 (m, 2H), 7.60 (d, *J* = 7.8 Hz, 2H), 8.15 (d, *J* = 7.8 Hz, 2H). ^13^C-NMR (CDCl_3_) δ, ppm: 20.4, 27.0, 30.2, 36.5, 39.3, 43.7, 68.0, 109.7, 113.1, 119.1, 119.6, 122.6, 123.0, 124.1, 124.2, 125.7, 128.2, 129.0, 132.9, 137.1, 150.9, 164.5. HRMS *m/z* (M+H)^+^: Calcd for C_44_H_45_N_6_O_2_, 689.3599; found: 689.3608. 

*N1,2-Bis-((2-evodiamin-N13-yl)ethyoxy)ethane* (**3-3**). Solid product 0.86 g, yield 46.0%, m.p. 143–145 °C. ^1^H-NMR (CDCl_3_) δ, ppm: 2.30 (s, 6H), 2.78–2.93 (m, 4H), 3.05–3.16 (m, 2H), 3.18–3.19 (m, 4H), 3.44–3.52 (m, 4H), 4.07–4.13 (m, 2H), 4.45–4.52 (m, 2H), 4.76–4.82 (m, 2H), 5.97 (s, 2H), 7.08–7.12 (m, 6H), 7.17–7.23 (m, 4H), 7.37–7.39 (m, 2H), 7.51 (d, *J* = 7.8 Hz, 2H), 8.05 (d, *J* = 7.8 Hz, 2H). ^13^C-NMR (CDCl_3_) δ, ppm: 20.3, 36.5, 39.3, 43.7, 67.9, 70.0, 70.8, 109.8, 113.1, 118.9, 119.7, 122.5, 122.6, 123.1, 124.1, 125.8, 128.9, 129.0, 132.9, 137.2, 150.9, 164.6. HRMS *m/z* (M+H)^+^: Calcd for C_44_H_45_N_6_O_4_, 721.3497; found: 721.3489.

### 3.4. Synthetic Procedure for **4-1**, **4-2**, **5**, **6**, **6-1** and **6-2**

NaOH–H_2_O (20%, 10.0 mmol) was added into a solution of 2.0 mmol of the appropriate compound **2-19** or **2-20** and **2-23** in ethanol (30 mL) and the mixture was heated to reflux for 3–4 h, while monitored by TLC. The mixture was neutralized with 20% HCl, the ethanol was removed under reduced pressure. The residue was extracted with EtOAc, and after the EtOAc was removed under reduced pressure, the crude product was purified by column chromatography to afford the pure target compounds.

*N13-(2-Hyroxyethyl)evodiamine* (**4-1**). Colorless solid product 0.43 g, yield 62.0%, m.p. 166–168 °C. ^1^H-NMR (DMSO-*d_6_*) δ, ppm: 2.38 (s, 3H), 2.71–2.83 (m, 1H), 2.95–3.02 (m, 1H), 3.03–3.21 (m, 1H), 3.64–3.69 (m, 2H), 4.30–4.43 (m, 2H), 4.66 (dd, *J*_1_ = 12.3 Hz, *J*_2_ = 3.3 Hz, 1H), 4.87 (t, *J* = 5.4 Hz, 1H), 6.18 (s, 1H), 7.04–7.09 (m, 1H), 7.16–7.22 (m, 2H), 7.30 (d, *J* = 8.1 Hz, 1H), 7.50–7.57 (m, 3H), 7.91 (dd, *J*_1_ = 8.4 Hz, *J*_2_ = 1.2 Hz, 1H). ESI-MS, *m*/*z* 348.2 [M+H]^+^. Anal. Calcd for C_21_H_21_N_3_O_2_: C, 72.60; H, 6.09. Found: C, 72.56; H, 6.17.

*N13-(3-Hyroxypropyl)evodiamine* (**4-2**). Yellowish solid product 0.51 g, yield 70.6%, m.p. 82–84 °C. ^1^H-NMR (CDCl_3_) δ, ppm: 2.10–2.16 (s, 2H), 2.45 (s, 3H), 2.90–2.94 (m, 1H), 3.03–3.07 (m, 2H), 3.13–3.17 (m, 1H), 3.66 (d, *J* = 5.1 Hz, 2H), 4.32–4.36 (m, 1H), 4.45–4.52 (m, 1H), 4.89–4.93 (m, 1H), 6.08 (s, 1H), 7.21–7.34 (m, 4H), 7.48–7.51 (m, 2H), 7.62–7.66 (m, 1H), 8.13 (d, *J* = 4.2 Hz, 1H). ^13^C-NMR (CDCl_3_) δ, ppm: 20.4, 32.4, 36.4, 39.5, 40.3, 59.0, 68.1, 110.2, 113.6, 119.1, 119.8, 122.8, 123.1, 124.1, 124.8, 125.8, 128.5, 129.0, 133.1, 137.4, 151.0, 164.5. HRMS *m/z* (M+H)^+^: Calcd for C_22_H_24_N_3_O_2_, 362.1863; found: 362.1876.

*4-(Evodiamin-N13-yl)butyric acid* (**5**). Colorless solid product 0.56 g, m.p. 234 °C (decomposed) yield 69.5%. ^1^H-NMR (DMSO-*d_6_*) δ, ppm: 1.97–2.01 (m, 2H), 2.29 (t, *J* = 7.2 Hz, 2H), 2.36 (s, 3H), 2.79–2.82 (m, 1H), 3.00–3.07 (m, 1H), 3.10–3.14 (m, 1H), 4.24–4.31 (m, 2H), 4.68 (dd, *J*_1_ =12.6 Hz, *J*_2_ = 3.9 Hz, 1H), 6.15 (s, 1H), 7.08–7.13 (m, 1H), 7.20–7.26 (m, 2H), 7.32 (d, *J* = 7.8 Hz, 1H), 7.53–7.61 (m, 3H), 7.94 (d, *J* = 6.6 Hz, 1H), 12.2 (s, 1H, –CO_2_H). ^13^C-NMR (CDCl_3_) δ, ppm: 19.8, 24.8, 30.8, 36.0, 42.6, 57.2, 67.1, 110.1, 112.2, 118.8, 119.2, 122.2, 123.0, 123.5, 125.3, 128.0, 128.4, 132.9, 136.8 150.6, 163.6, 174.0. HRMS *m/z* (M-H)^-^: Calcd for C_23_H_22_N_3_O_3_, 388.1667; found: 388.1670.

*2-(Evodiamin-N13-yl)acetaldehyde* (**6**). This compound was obtained by the hydrolysis of 9-(2,2-diethoxyethyl)evodiamine (**2-18**). Into a solution of **2-18** (1.26 g, 3.0 mmol) in ethanol (20 mL) was added 10% hydrochloric acid (10 mL), and the mixture was stirred for 4-5 h at room temperature, while monitored by TLC. After the reaction was complete, the mixture was neutralized with 10% NaOH, and the ethanol was evaporated under reduced pressure. The product precipitated when the mixture was cooled to room temperature, and the product **6** (0.35 g) was collected by filtration. Yield 33.8%, m.p. 149–151 °C. ^1^H-NMR (CDCl_3_) δ, ppm: 2.47 (s, 3H), 2.89–3.01 (m, 1H), 3.03–3.10 (m, 1H), 3.31–3.38 (m, 1H), 4.90–4.99 (m, 2H), 5.12–5.21 (m, 1H), 5.95 (s,1H), 7.15–7.20 (m, 1H), 7.21–7.25 (m, 2H), 7.27–7.32 (m, 1H), 7.32–7.37 (m, 1H), 7.42–7.48 (m, 1H), 7.49–7.56 (m, 1H), 7.62 (t, *J* = 6.6 Hz, 1H), 9.88 (s, 1H). HRMS m/z (M+H)^+^: Calcd for C_21_H_20_N_3_O_2_, 346.1550; found 346.1549.

*2-(Evodiamin-N13-yl)acetaldehyde oxime* (**6-1**). Compound **6-1** was obtained by the condensation reaction of **6** with hydroxylamine hydrochloride. Into a solution of **6** (0.52 g, 1.5 mmol) in absolute ethyl alcohol (20 mL) was added hydroxylamine hydrochloride (0.21 g, 3.0 mmol) and triethylamine (0.61 g, 6 mmol) and the mixture was stirred for 4-5 h at room temperature, while monitored by TLC. After the reaction was complete the ethanol was evaporated under reduced pressure. The product **6-1** (0.30 g) was obtained by filtration, yield 27.8%, m.p. 201–203 °C. ^1^H-NMR (CDCl_3_) δ, ppm: 2.42 (s, 3H), 2.87–2.98 (m, 1H), 3.01–3.10 (m, 1H), 3.16–3.27 (m, 1H), 4.89–4.98 (m, 2H), 5.11–5.20 (m, 1H), 5.33–5.40 (m, 1H, -OH), 5.95 (s, 0.48H), 5.98 (s, 0.52H), 7.15–7.20 (m, 1H), 7.21–7.25 (m, 2H), 7.27–7.32 (m, 1H), 7.32–7.37 (m, 1H), 7.42–7.48 (m, 1H), 7.49–7.56 (m, 1H), 7.62 (t, *J* = 6.6 Hz, 1H), 8.14 (d, *J* = 7.8 Hz, 1H). HRMS *m/z* (M+H)^+^: Calculated for C_21_H_21_N_4_O_2_, 361.1659; found 361.1670.

*2-(Evodiamin-N13-yl)acetaldehyde O-methyl oxime* (**6-2**). Compound **6-2** was synthesized as **6-1**, yield 57.0%, m.p. 216 °C (decomposed). ^1^H-NMR (CDCl_3_) δ, ppm: 2.41 (s, 3H), 2.89–2.97 (m, 1H), 3.02–3.07 (m, 1H), 3.17–3.27 (m, 1H), 3.86 (s, 2H, -OCH_3_), 3.99 (s, 1H, -OCH_3_), 4.88–4.92 (m, 1H), 4.94–4.96 (m, 1H), 5.04–5.14 (m, 1H), 5.93 (s, 0.34H), 5.98 (s, 0.66H), 7.18–7.23 (m, 2H), 7.24–7.26 (m, 1H), 7.28–7.34 (m, 2H), 7.44–7.48 (m, 1H), 7.48–7.53 (m, 1H), 7.59–7.64 (m, 1H), 8.15 (d, *J* = 0.9 Hz, 1H). ESI-MS, *m*/*e* 375.2 [M+H]^+^. Anal. Calcd for C_22_H_22_N_4_O_2_: C, 70.57; H, 5.92. Found: C, 70.60; H, 6.07.

### 3.5. Solubility of N13-Substituted Evodiamine Derivatives in SGF and SIF Solutions

SGF and SIF solutions were prepared as described in the United States Pharmacopoeia [31], and the solubilities of N13-substituted evodiamine derivatives in SGF and SIF solutions were determined by the reported method [32,33,34,35] with slight modifications.

### 3.6. Inhibitory Effects of N13-Substituted Evodiamine Derivatives toward Six Cultured Human Cancer Cell Lines

The growth inhibition effects of the thirty N13-substituted evodiamine derivates on human prostate cancer cell lines (DU-145 and PC-3), lung cancer cell line (H460), breast cancer cell line (MCF-7), colon cancer cell line (HCT-5) and glioblastoma cell lines (SF-268) were determined by MTT assay with evodiamine as positive control. 

### 3.7. Apoptosis of Human Cancer Cells Induced by **2-2**, **2-3**, **2-26** and **3-2**

Apoptosis of human cancer cell lines (MCF-7, NCl-H460 and HCT-15) induced by **2-2**, **2-3**, **2-16** and **3-2** was determined by flow cytometry analysis using the Annexin V-FITC/PI Apoptosis Kit. Cancer cells were plated at a density of 3 × 10^5^ cells per well in 6-well plates, incubated overnight and then treated with different concentrations of test compounds at their respective IC_50_ levels (predetermined by the MTT cytotoxicity assays) for 48 h. Staining was performed according to the manufacturer’s instructions. Stained cells were analyzed by a FACSCalibur flow cytometer (Becton Dickinson) using the CellQuest™ software. The percentages of early apoptosis were calculated by Annexin V-positivity and PI-negativity, while that of late apoptosis were determined by Annexin V-positivity and PI-positivity.

## 4. Conclusions

In conclusion, we have synthesized thirty-eight evodiamine derivates with different substituents at the N13-position and preliminarily evaluated their inhibitory activities against six cancer cell lines and their solubility in SGF and SIF. It could be concluded that: (1) the appropriate substitution on the N13-position of evodiamine made some derivatives (compounds **2-2**, **2-3**, **2-9**, **2-16**, **3-2**) show more antitumor potency and broadened antitumor spectra. Among these, compound **2-16** displayed the highest antitumor activity and a broader spectrum, with IC_50_ values of less than 2 µM; (2) their solubility was obviously improved; (3) compounds **2-3**, **2-16** and **3-2** had a significant impact inducing apoptosis in some cancer cell lines. Meantime, the preliminary structure-activity relationships of these derivatives were discussed. Further investigation of these derivatives, including more screening of anticancer activity and determination of their mechanisms of action against tumor cell lines are ongoing.
